# Contralateral Spread of Asymmetrical Tremor in Parkinson's Disease

**DOI:** 10.1002/mdc3.70353

**Published:** 2025-09-16

**Authors:** Jacopo Pasquini, Nicola Pavese, Roberto Ceravolo, Rick C. Helmich, Günther Deuschl

**Affiliations:** ^1^ Department of Clinical and Experimental Medicine University of Pisa Pisa Italy; ^2^ Translational and Clinical Research Institute, Faculty of Medical Sciences, Newcastle University Newcastle upon Tyne UK; ^3^ Department of Nuclear Medicine and PET Centre Institute of Clinical Medicine Aarhus University Aarhus Denmark; ^4^ Center for Neurodegenerative Diseases‐Parkinson's Disease and Movement Disorders, Neurology Unit, Azienda Ospedaliero‐Universitaria Pisana Pisa Italy; ^5^ Donders Centre for Cognitive Neuroimaging, Donders Institute for Brain, Cognition and Behaviour, Radboud University Nijmegen The Netherlands; ^6^ Department of Neurology and Center of Expertise for Parkinson & Movement Disorders Donders Institute for Brain, Cognition and Behaviour, Radboud University Medical Center Nijmegen The Netherlands; ^7^ Department of Neurology Universitätsklinikum Schleswig‐Holstein, Kiel Campus, and Christian‐Albrechts‐University Kiel Germany

**Keywords:** tremor, Parkinson's disease, laterality of tremor, disease progression, clinical assessment

## Abstract

**Background:**

Parkinsonian tremor usually starts asymmetrically. The mid‐term prognosis of this lateralized tremor is unknown, as is the development of tremor in the contralateral arm.

**Objective:**

To investigate the occurrence of contralateral tremor in the Parkinson's Progression Marker Initiative database, with data available for 7 years.

**Methods:**

Tremor requiring treatment (TRT) was defined as any rest, postural or kinetic tremor with amplitude >1 cm (MDS‐UPDRS score ≥2) as this criterion is commonly accepted as an insufficiently treated tremor. Tremor was analyzed by side mainly in the off‐medication state.

**Results:**

At baseline, 348 (87.7%) of the 397 patients with Parkinson's disease had tremor at least on one side of the body. 183 (46%) had only mild tremors but 165 (41.6%) had TRT. 159 patients (40.1%) had lateralized TRT and six (1.6%) had bilateral TRT. Among patients with asymmetrical TRT, 40 patients (25.2%) developed contralateral TRT at 3 years, 49 patients (30.8%) at 5 years, and 61 patients (38.4%) at 7 years. The side more affected by tremor was also more affected by other cardinal signs. In 159 patients with initially asymmetrical TRT, tremor severity did not increase on the more tremulous side over the 7‐year period. However, there was an increase in tremor on the contralateral side. This was associated with a clear increase in bradykinesia and rigidity on both sides.

**Conclusion:**

The study findings may prove beneficial in counseling patients with TRT, and may also provide an explanation as to why the worsening of tremor is not correlated with overall disease progression.

Tremor is the eponymous sign of the shaking palsy and is yet poorly understood in many respects. Particularly, the long‐term course of this troublesome and disabling symptom has only been studied for relatively short periods.^1^ A distinctive feature is the asymmetrical onset of PD,^2^ and particularly the classic resting tremor, which can facilitate the neurologist's overall clinical diagnosis. Asymmetrical tremor may progress to the contralateral side but evidence regarding the timing and severity of this phenomenon is lacking. With the focus on early manifestations of PD, studies on the natural course are now increasingly popular, but their focus has only rarely been on tremor.^3^, ^4^ Particularly, the question for the “time‐to‐contralateral tremor” has to our knowledge never been studied. In the context of tremor treatment, this is becoming an increasingly pertinent question, given the availability of invasive treatments for PD manifestations on a single body side, such as Magnetic Resonance guided Focused Ultrasound (MRgFUS) and in some cases deep brain stimulation. Indeed, it is not known if and when the second side will be affected in patients with asymmetrical tremor. Here we address this question by analyzing the cohort of patients from the Parkinson Progression Marker Initiative^5^ who have been assessed prospectively on an annual basis using the MDS‐UPDRS. The main research question is when and to what extent functionally impairing tremor develops on the second side after a relevant tremor has been diagnosed on the first side. Using the anchors defined by the MDS‐UPDRS, we define a tremor severity >1 cm at the fingertips under resting or postural or kinetic conditions as “tremor requiring treatment” (TRT).

## Methods

### Participants

This paper uses the clinical database of the Parkinson Progression Marker Initiative (PPMI).^5^ The data includes demographic and clinical characteristics of 397 sporadic PD participants, also included in a previous analysis from our group.^4^ This analysis used data from the PPMI cohort enrolled between 2010 and 2020. Protocol information for The Parkinson's Progression Markers Initiative (PPMI) 001 AM 12 can be found at the following link: https://www.ppmi-info.org/study-design/archive-of-research-docs-and-sops.html. The PPMI was designed to be an 8‐year natural history study (with a minimum of 5‐year involvement) of de novo idiopathic PD participants. All PD subjects were planned to have an annual assessment of the motor exam in a practically defined off‐state and a repeat on‐state assessment 1 h after receiving their usual PD medication in clinic.

For this study, we considered the first 7 years of follow up after enrollment. Since not all patients attended their OFF‐state assessment each year, in Table S1A we show the number of participants that attended each follow up. The clinical characteristics of tremor, i.e., its occurrence on the more‐ and less‐affected sides, as well as their progression over time, are described in the OFF‐state.

Furthermore, because patients entering the PPMI were untreated and treatment was introduced after at least 6 months following enrollment according to participants’ clinical needs, not all subjects included in this analysis were assessed in the ON‐state at every assessment. Indeed, patients within the PPMI are asked to come for the assessment in the OFF‐medication state explaining why there are more assessments under OFF‐ than under ON‐conditions. In Table S1B we reported the number of participants with ON assessments at every follow up. A smaller part of the analysis presented in this paper focuses on the ON‐state tremor scores to investigate the development of clinically‐significant tremor on the initially less‐affected side (see below “On assessments” section).

The clinical data of the cohort at baseline are summarized in Tables 1, 2.

**TABLE 1:** Baseline clinical characteristics of the PPMI cohort included in this study

	Baseline characteristics
Number of eligible participants	397
Males/Females	261/136 (66%/34%)
Age at diagnosis (years), mean (SD), range	61.91 (9.59), 34–85
Disease duration at enrollment (months), mean (SD), range	6.76 (4.07), 0–37
MDS‐UPDRS III, mean (SD), range	21.00 (8.88), 4–51
Bradykinesia score^a^, mean (SD)	8.32 (4.90)
Rigidity score^b^, mean (SD)	3.82 (2.66)
Hoehn & Yahr, median (range)	2 (1–2)

^a^ Sum of MDS‐UPDRS of subitems of items 3.4, 3.5, 3.6, 3.7, 3.8 (Score range 0–40);

^b^ Sum of subitems of item 3.3 (Score range 0–20); Abbreviations: MDS‐UPDRS III = Movement Disorders Society—Unified Parkinson Disease Rating Scale part III; SD = standard deviation.

### Definition of Tremor Requiring Treatment (TRT)

Within the PPMI database, tremor is measured with the MDS‐UPDRS. Lateralized items related to tremor come only from the physician exam of part III (motor score) covering information for postural tremor (Item 3.15), kinetic tremor (3.16), and rest tremor (3.17) for the upper extremity, and for the lower extremity for rest tremor only (3.17). All these tremor manifestations are measured with the following anchors documented as the maximal amplitude during the clinical exams. 0: No tremor. 1: Slight: < 1 cm. 2: Mild: > 1 cm but <3 cm. 3: Moderate: 3–10 cm. 4: Severe: > 10 cm.

The further tremor items 3.17e (rest tremor amplitude‐lip/jaw) and 3.18 (constancy of rest tremor) are excluded from this analysis as they cannot contribute to the definition of lateralized tremor.

For the purpose of this study, we define a tremor possibly eligible for treatments as having an amplitude of more than 1 cm at fingertips (corresponding to the anchor score of 2 in the upper limbs tremor items) and call this a “tremor requiring treatment (TRT).” Such TRT is assumed if at least one of the upper limbs tremor scores for rest, postural and kinetic tremor is rated as 2 or more. This criterion is commonly used as an inclusion criterion for studies testing invasive interventions such as MRgFUS for tremor.^6^, ^7^, ^8^, ^9^

### Definition of Lateralized TRT

The TRT criterion allows the identification of participants with a lateralized TRT (i.e., an upper limb tremor subitem score ≥2, with contralateral upper limbs subitems scores ≤1), a bilateral TRT (an upper limb tremor subitem score ≥2 both on right and left sides), and participants without TRT (all upper limbs tremor subitems scores ≤1).

### Definition of Lateralized Scores

A total lateralized tremor score was also calculated by summing the lateralized MDS‐UPDRS 3.15, 3.16 and 3.17 subitems. Here, we also included the rest tremor lower limb score in the sum, as this contributes to the overall burden of tremor score on each side. Therefore, the lateralized tremor score ranges between 0 and 16 points. The initial side where total tremor score was higher was defined as the “dominant side of tremor,” the opposite side was defined as the “non‐dominant side of tremor.”

Additionally, for comparison, we have calculated the lateralized scores for bradykinesia and rigidity. The lateralized bradykinesia score was calculated as the sum of right or left subitems 3.4, 3.5, 3.6, 3.7 and 3.8 (score range 0 to 20). The lateralized rigidity score was calculated as the sum of right or left subitems 3.3 (score range 0 to 8), respectively.

### Definition of Contralateral Side Tremor Development

Each patient had multiple assessments on the MDS‐UPDRS over the 7‐years span within the database. Since not all patients attended all the 7‐year assessments, by convention, we defined a patient with a tremor fulfilling our contralateral TRT‐criterium to have TRT for the rest of the observation period.

### On‐Assessments

All preceding methodological details refer to the participant in the off‐state. Where, available, we also investigated the ON‐state to assess the presence of contralateral tremor after taking the usual PD treatment.

As described in the introductory paragraph of the Methods section, the ON state evaluation was available only for a subset of participant after the 1‐year follow up (Table S1B). Therefore, in the ON‐state we could only assess the cumulative number and percentage of participants with contralateral tremor on the total number of single participants that attended at least one follow up. More practically, 134 of 159 total participants with lateralized tremor had at least one ON‐state assessment over the entire follow up period. In these 134, 83 showed a right‐dominant tremor and 51 showed a left‐dominant tremor. Similarly to the OFF‐state, in the ON‐state we defined a patient with a tremor fulfilling our TRT‐criteria to have TRT for the rest of the observation period.

### Statistical Analysis

Descriptive categorical data is reported as numbers and percentages, while continuous scores are reported as mean and standard deviation (SD).

To investigate the development of TRT on the contralateral side, the Kaplan–Meier method was employed to generate the probability graph and associated descriptive tables.

To assess whether there was a significant progression over time of tremor, bradykinesia and rigidity lateralized scores (on the more and less affected sides by tremor), linear mixed‐effect models fit by restricted maximum likelihood with random intercept were employed. Time (as a continuous variable), sex, age and disease duration at enrollment were used as predictors, as shown in the following equation:

$$\text{dominant}/\text{nondominant tremor}/\text{rigidity}/\text{bradykinesia scores}\sim \text{time}+\mathrm{sex}+\mathrm{age}\;\mathrm{at}\;\text{diagnosis}+\text{disease duration}\;\mathrm{at}\;\text{enrollment}+\left(1|\mathrm{id}\right)$$

Statistical analyses were conducted in SPSS Statistics version 29 (IBM®, https://www.ibm.com/products/spss-statistics, RRID: SCR_002865).

### Data Sharing

Data used in the preparation of this article was obtained on December 4, 2024 from the Parkinson's Progressive Markers Initiative (PPMI) database (https://www.ppmi-info.org), RRID:SCR_006431. For up‐to‐date information on the study, visit www.ppmi‐ info.org. This analysis used data openly available from PPMI (Tier 1 Data). Codes generated to perform the analyses in this article are shared on Zenodo at the following DOI: https://doi.org/10.5281/zenodo.14892610.

## Results

### Cohort Description

The PPMI cohort included in this study consists of 397 patients at baseline. We considered a maximum of 7 years follow‐up for these participants. Demographic and basic clinical characteristics over the 7 years are reported in a previous study from our group,^3^ while in Table 1 we summarize the main characteristics at baseline.

**Figure 1:**
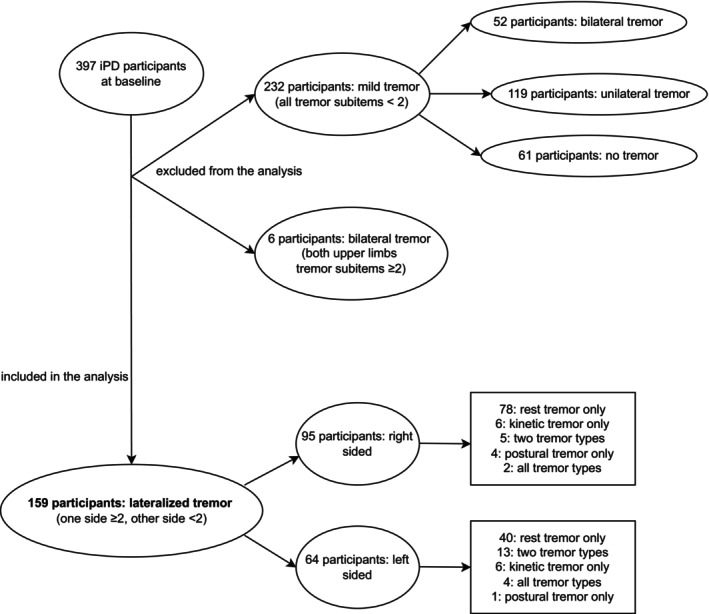
Flowchart illustrating participants subgrouping and inclusion in the analysis in regards to tremor scores, types, and laterality.

At baseline, out of the cohort of 397 patients, 159 participants (40.1% of the total cohort) showed TRT in the upper limbs, (Table 2, Fig. 1). Of these, 95 showed a right‐dominant tremor and 64 a left‐dominant tremor. The majority of these 159 participants showed rest tremor (138 subjects accounting for 86.8%, 85 right‐sided, 53 left‐sided). Twenty‐six subjects also showed lateralized postural tremor (11 right‐sided, 15 left‐sided) and 27 subjects also showed lateralized kinetic tremor (10 right‐sided, 17 left‐sided). It should be noted that rest, postural and kinetic tremors may coexist in the same participants therefore the total number of the precedingly described tremor types is not reflective of the single participants with lateralized tremor. Accordingly, in 24 participants a combination of rest, postural and/or kinetic tremor was present (Fig. 1, Table S2). Notably, for all participants with more than one type of tremor, the different types of tremor were lateralized on the same side.

**TABLE 2:** Number of participants with no tremor, lateralized tremor, and bilateral tremor at baseline. This classification allows each patient to be classified into only one tremor group according to MDS‐UPDRS tremor items 3.15, 3.16, 3.17 for rest, postural and kinetic tremor

Severity of tremors (worst tremor of the three tremor types—rest, postural and kinetic)	Number of subjects (total number: 397)
Patients with absent or slight tremor (both sides tremor <2)	232 (58.4%)
Consisting of:	
1. patients with bilateral slight tremors (on both sides at least one tremor = 1)	52 (22.4%/13.0%^a^)
2. patients with lateralized slight tremors (one side at least one tremor = 1, other side all tremors = 0)	119 (51.3%/30.0%^a^)
3. patients without tremor bilaterally (both sides = 0)	61 (26.3%/15.4%^a^)
Lateralized TRT (one side ≥2, other side <2)^a^	159 (40.1%)
Bilateral TRT (both sides ≥2)^a^	6 (1.5%)

^a^ Percentage based on patients with absent or slight tremor (232) / percentage based on the total (397) patients.

Abbreviation: TRT, tremor requiring treatment.

Lower limbs tremor was present in 79 of the 397 PD participants (19.9%) and was right‐sided in 45 participants and left‐sided in 34. In 159 participants with lateralized upper limbs TRT, lower limbs tremor was present in 38 participants (Fig. S1).

### Tremor Occurrence on the Contralateral Side

**Figure 2:**
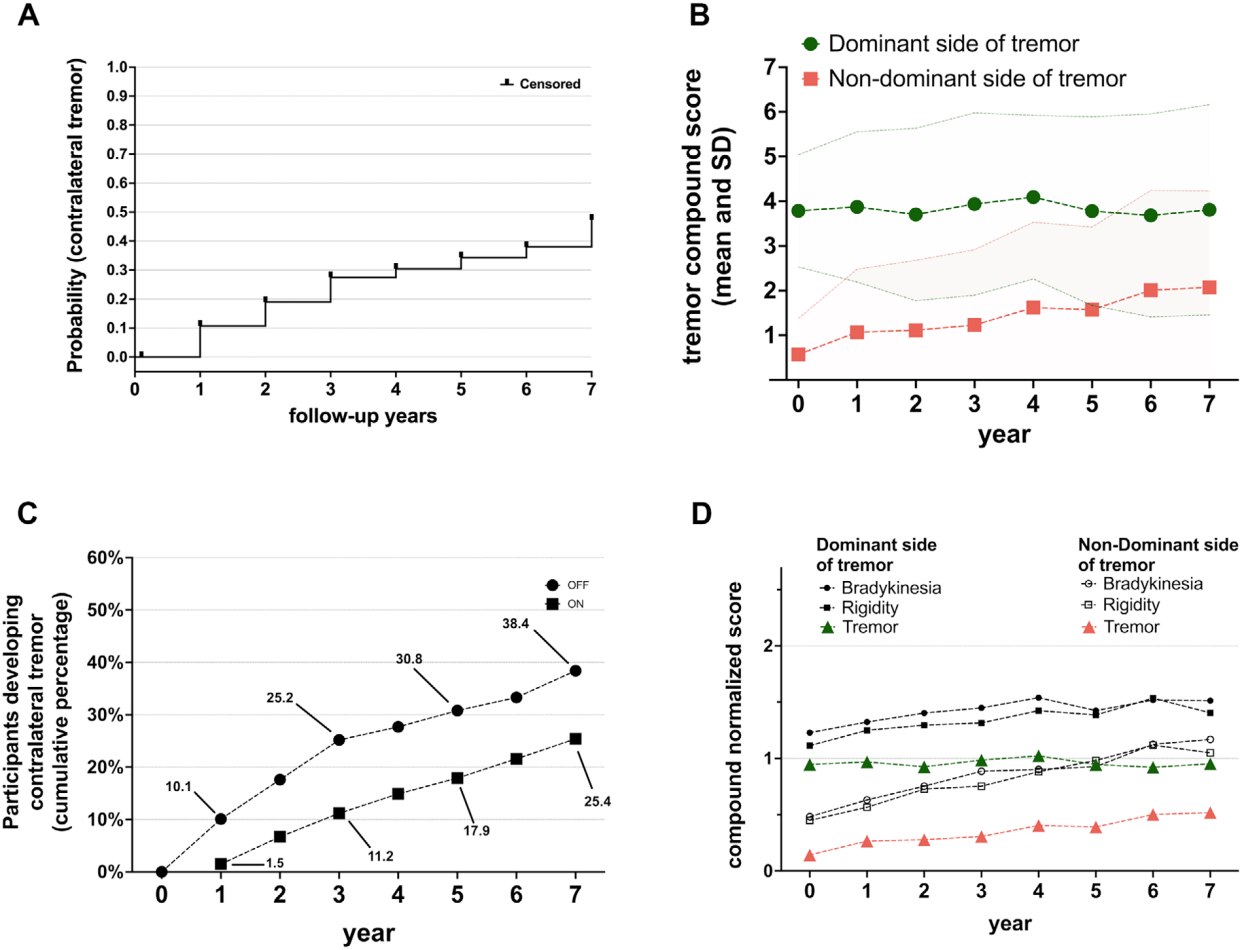
(A) represents the probability function for the development of contralateral TRT (ie, a score equal or greater than two in any of the tremor subitems of MDS‐UPDRS items 3.16, 3.17 or 3.18) estimated with the Kaplan–Meier method. (B) represents the tremor severity (mean ± SD) of the OFF‐state lateralized compound tremor score for postural, kinetic and rest tremor. Dominant versus non‐dominant side of tremor were merged for all 159 patients. (C) shows the cumulative percentage of patients developing TRT over the 7 years period comparing OFF‐state (considering the initial 159 participants with lateralized tremor) and ON‐state assessments (considering the subgroup of 134 participants with lateralized tremor that attended at least one ON‐state assessment during follow up). (D) shows the mean (standardized to a 0 to 4 scale by dividing the total scores by the number of summed items) lateralized scores for bradykinesia, rigidity and tremor in the 159 lateralized TRT patients for each side separately at each available follow up visit. Linear mixed model showed that all compound scores worsen except for the tremor on the initially affected side.

Of the 159 patients who had asymmetrical TRT at baseline, cumulatively 25.2% developed a TRT on the second side at 3 years, 30.8% at 5 years and 38.4% at 7 years. Figure 2A represents the probability function of developing contralateral TRT in the examined group estimated with the Kaplan–Meier method; Table S3 shows the associated survival table. In 61 patients that developed contralateral tremor, the mean time to event is 3.23 years (median 3.0) and SD 2.09. Rest tremor affected the majority of these 159 participants with lateralized tremor and was by far the most common type of tremor (Fig. 1, Table S2).

The development of contralateral TRT was also examined in the ON‐state. Due to the study design of the PPMI (participants initially unmedicated, off‐assessment in clinic followed by ON‐assessment if participant is treated), we were only able to assess how many participants cumulatively developed a TRT in ON‐state. Of the 159 participants with baseline lateralized TRT, 134 (83 right‐dominant tremor and 51 were left‐dominant tremor) attended at least one follow up on‐assessment. Table S1B shows how many participants attended the ON state assessment at each follow up. Figure 2C represents the ON (and OFF) cumulative percentage of participants who developed contralateral TRT. 25.4% of 134 participants after 7 years showed a contralateral TRT, compared to 38.4% for the off‐assessments.

Finally, we analyzed the progression of tremor, rigidity and bradykinesia compound scores separately for the dominant side of tremor and non‐dominant side of tremor (dominance was defined by the side with greater tremor at baseline) (Fig. 2B,D). Interestingly the compound tremor score increased only on the initially non‐affected side but the rigidity and bradykinesia scores worsened significantly on both sides (Table 3 and S4).

**TABLE 3:** Results of the linear mixed effects models to test the effect of time, sex, age at diagnosis, disease duration at enrollment on bradykinesia, rigidity and tremor scores on the dominant and non‐dominant sides of tremor in 159 participants with tremor requiring treatment (TRT). Of note, the dominant side of tremor was the side initially more affected with tremor

	Bradykinesia		Rigidity		Tremor	
	Dominant side	Non dominant side	Dominant side	Non dominant side	Dominant side	Non dominant side
Follow‐up time, estimate (SE)	0.247 (0.058)***	0.513 (0.055)***	0.098 (0.026)***	0.183 (0.024)***	−0.008 (0.036)	0.205 (0.028)***

*Note*: The variable “Time” refers to follow‐ups, once every year after baseline. The variable “Sex” was coded as 0 for females and 1 for males. Age and disease duration are considered at enrollment. Significance level: * *p* < 0.05; **p < 0.01; ***p < 0.001.

### TRT Occurrence in Participants with Mild Lateralized Tremor

Among 232 PD participants with mild (all tremor subitems <2) or no tremor at baseline, 119 showed mild lateralized tremor (one tremor subitem =1 on one side, all tremors subitems = 0 on the opposite side; upper limbs only). In this group, the probability (Kaplan–Meier method) of developing TRT on the ipsilateral side was 70% at 7 years and 26% on the contralateral side (Fig. S2).

## Discussion

The lateralized progression of tremor in Parkinson's disease has not been studied, although it is a clinically highly important information for invasive procedures, especially if initially directed to a single‐hemisphere (unilateral deep brain stimulation or MR‐guided focused ultrasound). This study shows that among the 159 patients in the PPMI cohort with significant, lateralized tremor (asymmetrical TRT), the contralateral side also developed TRT in 30% of cases at 5 years and almost 40% of cases at 7 years. These data are notable for patient counseling. Additionally, in this group we show that the dynamics of tremor worsening differ for the initially more affected side from the second affected side. This does not only apply to TRT but also mild tremors: these show a more pronounced deterioration in the initially affected side compared to the less rapid decline of the less affected side, confirming the continuous development of lateralization of tremor into the more advanced stages. Interestingly, almost all 159 participants with lateralized TRT had a rest tremor, while only a minority had lateralized action tremor. Conversely, 232 participants had tremor scores <2 in both upper limbs. It is possible that action tremor is indeed less represented at this stage of the disease, although another possibility is that action tremor amplitude continues to increase after 10 seconds of posturing, which is the temporal limit to observe postural tremor in the MDS‐UPDRS item 3.15.^10^

In our study, 87.7% of patients, diagnosed at an early stage, had some kind of pathologic tremor manifestation at baseline.^2^ This is a high number and may reflect the fact that PD is suspected by non‐specialists earliest when presenting with tremor, and subsequently these patients are more likely to be referred to specialized movement disorders clinics which recruited the PPMI cohort. This cohort was diagnosed at an early stage of the disease of only 6 months. One of the characteristics of classic Parkinsonian tremor is that it starts mostly asymmetrically. This is also true of other core signs of Parkinson's disease. For the formerly used British Brain Bank criteria^11^ for the diagnosis of PD, the asymmetrical manifestation of the cardinal signs was even a core diagnostic criterion. In the entire PPMI group of 397 participants, 70% had an asymmetrical tremor with 40% severe enough to be classified as TRT.

A critical methodological issue of this study is the definition of TRT. We consider a tremor with an amplitude at the fingertips below 1–2 cm being less disturbing for the patient than a tremor above this amplitude. This is based mainly on clinical experience, because treatments are usually only considered successful if they reduce the tremor amplitude below this threshold.^12^ Consistent with this, tremor amplitudes above 1–2 cm are used as a threshold criterion for inclusion into MRgFUS studies.^9^ The amplitude threshold below which surgery is no longer an option may vary by center and patient. Our chosen threshold is pragmatic also considering the experience‐based definition of the scale anchors between “slight” and “moderate.” In our view, it is not critical which tremor type (rest, posture, kinetic) is present in a particular patient, as all of them can be severely bothering.

To our knowledge, lateralization of tremor has not been analyzed in detail. While a different course of tremor progression on both sides may be clinically intuitive, the finding of a significant increase over time only in the contralateral side is somewhat unexpected. Indeed, in 159 participants with lateralized TRT, we found that mean tremor scores in this tremulous group do not further increase on the initially affected side during the first 7 years of disease. Conversely, bradykinesia and rigidity show significant increases on both sides. Of course, the lack of significant worsening of ipsilateral tremor average scores is influenced by the peculiar selection of participants in this analysis, which was tailored to understand the progression in PD patients with a lateralized tremor onset. This finding may contribute to explain why earlier studies on tremor progression did not find a worsening.^13^ A recent study in a large cohort of early to mid‐stage PD found that while overall bradykinesia and rigidity scores worsened over 2 years, rest tremor severity did not change significantly, while action tremors even decreased.^14^ The worsening of the overall tremor scores in the group is therefore mainly due to the worsening of the initially unaffected side developing tremor later. This was found despite similar worsening of bradykinesia and rigidity on both sides. The reason why tremor does not further increase from a certain time point on is not known. Different explanations come into play, two of them will be mentioned here: first, it might be a simple scale effect, as the anchors of the MDS‐UPDRS separate between tremors between below 1 cm, 1–3 cm, 3–10 cm and above 10 cm. The worsening could go in smaller steps and could only be detected with wearables. Second, the amplitude of tremor depends also on physical factors like the size and weight of the hand and obviously the maximum amplitude is clearly limited.

Progression of PD manifestations has been a topic over the past 40 years. While early evidence suggested that patients with predominant tremor have a slower overall disease progression and longer time to death^15^, ^16^, ^17^ this has meanwhile largely be replaced by the better prognostic value of non‐motor features.^18^ Tremor in Parkinson's disease is not related to a neuropathological lesion pattern.^19^ Functional parameters of presynaptic striatal function like measures of dopamine synthesis and storage (Fluoro‐Dopa‐PET) or labelling of presynaptic transport proteins (DAT SPECT) correlate nicely with bradykinesia and rigidity and even with the overall disease severity but not with tremor.^20^ The overall plateau effect of tremor scores, together with multiple other factors (eg, non‐dopaminergic neurotransmitter involvement), might explain some of these open questions.

Several limitations of this study should be acknowledged. This study has limited follow‐up time, but 7 years of follow‐up obtained from such a consistently recruited cohort is almost unique information. Secondly, the PPMI‐cohort may not be representative of the whole spectrum of early PD‐manifestations as tremulous patients may have been overrepresented, but this does not affect the present study dealing exactly with this subgroup. Since inclusion in the cohort was based on the baseline manifestation of an asymmetrical tremor, almost all participants included showed an asymmetrical rest tremor (see also Table 3). Therefore, in the analysis on the development of tremor on the initially non‐dominant tremor side we lumped together all three types of tremor. Future studies with larger groups of asymmetrical postural and kinetic tremor may attempt to investigate the contralateral spread specifically in these subtypes. In the analysis of the development of tremor on the initially less‐tremulous side, participants that developed a tremor amplitude score >1 in one the MDS‐UPDRS III tremor subitems were categorized as participants with a clinically significant tremor for the rest of the observation period. Since tremor is constantly changing in amplitude over time, we believe that this conceptual simplification was necessary to convey a clinically meaningful message. Nonetheless, we are aware that some PD patients may manifest tremor and then stop manifesting it. Therefore, a limitation of this study is also the inability to describe this phenomenon, which must be systematically investigated in a future study. Furthermore, in the current study we were unable to separate the postural and re‐emergent components of tremor upon posturing, since this study was based on the MDS‐UPDRS evaluation, which lumps together these two types of tremor in item 3.15. Finally, the responsiveness of tremor to dopaminergic medication was only analyzed relatively to the effect of treatment on the burden of TRT presentation (Fig. 2C). Future studies may investigate the responsiveness of tremor on the two sides in cases of bilateral tremor.

In conclusion, in this study we present data that may be clinically helpful for patients with asymmetrical tremor. Indeed, we have shown that 40% of patients have TRT on the initially less‐affected side after 7 years. Furthermore, we highlighted a lack of significant tremor progression in the initially more tremulous side, accompanied by a significant tremor score increase contralaterally. This finding contributes to explain the overall lack of tremor progression found in some previous studies.

## Author Roles

(1) Research project: A. Conception, B. Organization, C. Execution;

(2) Statistical Analysis: A. Design, B. Execution, C. Review and Critique;

(3) Manuscript: A. Writing of the first draft, B. Review and Critique.

JP: 1A, 1B, 1C; 2A, 2B; 3A.

N: 1A; 1B; 2A, 2C; 3B.

RC: 1B, 1C; 2A, 2C, 3B.

RCH: 1A; 2A, 2C; 3B.

GD: 1A, 1B; 1C; 2A; 2B; 3B.

## Disclosures

**Ethical Compliance Statement:** All participating PPMI sites received approval from an ethical standards committee prior to study initiation and written informed consent for research was obtained from all participants in the study. An additional specific IRB approval was not required to carry out the analysis presented in this paper. We confirm that we have read the Journal's position on issues involved in ethical publication and affirm that this work is consistent with those guidelines.

**Funding sources and Conflict of Interest:** The authors declare that there are no conflicts of interest relevant to this work. No specific funding was received for this work. PPMI—a public‐private partnership—is funded by the Michael J. Fox Foundation for Parkinson‘s Research and funding partners, including 4D Pharma, Abbvie, AcureX, Allergan, Amathus Therapeutics, Aligning Science Across Parkinson‘s, AskBio, Avid Radiopharmaceuticals, BIAL, BioArctic, Biogen, Biohaven, BioLegend, BlueRock Therapeutics, Bristol‐Myers Squibb, Calico Labs, Capsida Biotherapeutics, Celgene, Cerevel Therapeutics, Coave Therapeutics, DaCapo Brainscience, Denali, Edmond J. Safra Foundation, Eli Lilly, Gain Therapeutics, GE HealthCare, Genentech, GSK, Golub Capital, Handl Therapeutics, Insitro, Jazz Pharmaceuticals, Johnson & Johnson Innovative Medicine, Lundbeck, Merck, Meso Scale Discovery, Mission Therapeutics, Neurocrine Biosciences, Neuron23, Neuropore, Pfizer, Piramal, Prevail Therapeutics, Roche, Sanofi, Servier, Sun Pharma Advanced Research Company, Takeda, Teva, UCB, Vanqua Bio, Verily, Voyager Therapeutics, the Weston Family Foundation and Yumanity Therapeutics.

**Financial Disclosures of all authors (for the preceding 12 months):** JP: none. NP has participated in advisory boards (Britannia, Boston Scientific, Benevolent AI, Roche, Abbvie), has received honoraria from Britannia, Abbvie, GE Healthcare, Boston Scientific, Teva Pharmaceuticals and grants from Independent Research Fund Denmark, Danish Parkinson's disease Association, Parkinson's UK, Center of Excellence in Neurodegeneration (CoEN) network award, GE Healthcare Grant, Multiple System Atrophy Trust, Weston Brain Institute, EU Joint Program Neurodegenerative Disease Research (JPND), EU Horizon 2020 research and innovation programme, Italian Ministry of Health. RC has received congress speech honoraria from AbbVie, Zambon, Bial, General Electric, EverPharma and grants from the Italian Ministry of Health, Italian Ministry of Research, Tuscany Region, European Joint Programme‐Neuroscience Disease Research and the Fresco Foundation. GD has served as a consultant for Boston Scientific and Insightec. He receives royalties from Thieme publishers. He receives funding from the German Research Council (SFB 1261, T1) and private foundations. RH received funds for consultancy from Neurocrine Biosciences. RH received research grants from the MJ Fox Foundation, the Netherlands Brain Foundation, ParkinsonNL, and the Netherlands Organization for Health Research and Development.

## Supporting information


**TABLE S1.** Number of participants with a lateralized tremor at baseline involved at each follow up visit with the off‐state assessment (A) and on‐state assessment (B). The number of total participants is also split by right‐dominant and left‐dominant.
**TABLE S2.** Distribution of tremor types at baseline OFF‐condition in participants with a lateralized tremor.
**TABLE S3.** Survival table related to the development of contralateral upper limbs tremor score ≥2 in 159 participants with lateralized tremor at baseline.
**TABLE S4.** Results of the linear mixed effects models to test the effect of time, sex, age at diagnosis, disease duration at enrollment on bradykinesia, rigidity and tremor scores on the dominant and non‐dominant sides of tremor in 159 participants with tremor requiring treatment (TRT). The dominant side of tremor was the side initially more affected with tremor.
**Figure S1.** Diagram showing lower limb tremor distribution in the 397 idiopathic PD participants included in the study.
**Figure S2.** Probability functions (estimated with the Kaplan–Meier method) for the development of TRT (ie, a score equal or greater than two in any of the tremor subitems of MDS‐UPDRS items 3.16, 3.17 or 3.18) in 119 participants with mild lateralized tremor at baseline, ipsilaterally (left panel) and contralaterally (right panel).

## Data Availability

Data used in the preparation of this article was obtained on 2024‐04‐12 from the Parkinson's Progressive Markers Initiative (PPMI) database (https://www.ppmi-info.org), RRID:SCR_006431. For up‐to‐date information on the study, visit https://www.ppmi-info.org. This analysis used data openly available from PPMI (Tier 1 Data). Codes generated to perform the analyses in this article are shared on Zenodo at the following DOI: https://doi.org/10.5281/zenodo.14892610.
