# Supplementary material for: Contralateral Spread of Asymmetrical Tremor in Parkinson's Disease
Source: Mov Disord Clin Pract. 2025 Sep 16;13(3):702–10. doi: 10.1002/mdc3.70353 (PMC13042382; doi:10.1002/mdc3.70353)

**Supplementary material for:**

**Contralateral spread of unilateral tremor in Parkinson’s Disease**

*Jacopo Pasquini^1,2^, Nicola Pavese^2,3^, Roberto Ceravolo^1^, Rick Helmich^4,5^,*

*Günther Deuschl^6^*

**Supplementary Tables**

**Supplementary Table 1.** Number of participants with a lateralized tremor at baseline involved at each follow up visit with the off-state assessment (A) and on-state assessment (B). The number of total participants is also split by right-dominant and left-dominant.

**A.**

| **OFF** | Total participants, n | Right-dominant tremor participants, n | Left-dominant tremor participants, n |
| --- | --- | --- | --- |
| Baseline | 159 | 95 | 64 |
| Year 1 | 128 | 83 | 45 |
| Year 2 | 125 | 80 | 52 |
| Year 3 | 128 | 80 | 48 |
| Year 4 | 119 | 72 | 47 |
| Year 5 | 110 | 66 | 44 |
| Year 6 | 101 | 61 | 40 |
| Year 7 | 90 | 58 | 32 |

**B.**

| **ON** | Total participants, n | Right-dominant tremor participants, n | Left-dominant tremor participants, n |
| --- | --- | --- | --- |
| Baseline | 0 | 0 | 0 |
| Year 1 | 49 | 28 | 21 |
| Year 2 | 78 | 43 | 35 |
| Year 3 | 81 | 48 | 33 |
| Year 4 | 94 | 57 | 37 |
| Year 5 | 82 | 50 | 32 |
| Year 6 | 76 | 48 | 28 |
| Year 7 | 83 | 54 | 29 |

**Supplementary Table 2**. Distribution of tremor types at baseline OFF-condition in participants with a lateralized tremor.

| **Lateralized TRT** | **Dominant tremor type** | **N of subjects** |
| --- | --- | --- |
| **Right sided tremor (95 subjects)** | Kinetic only | 6 |
|  | Postural only | 4 |
|  | Rest only | 78 |
|  | Two types | 5 (3 KT+RT, 2 PT+RT) |
|  | All three types | 2 |
| **Left sided tremor (64 subjects)** | Kinetic only | 6 |
|  | Postural only | 1 |
|  | Rest only | 40 |
|  | Two types | 13 (4 KT+PT, 3 KT+RT, 6 PT+RT) |
|  | All three types | 4 |

*Abbreviations*. TRT, tremor requiring treatment.

**Supplementary Table 3.** Survival table related to the development of contralateral upper limbs tremor score ≥ 2 in 159 participants with lateralized tremor at baseline.

| Time point | BL | 1 | 2 | 3 | 4 | 5 | 6 | 7 |
| --- | --- | --- | --- | --- | --- | --- | --- | --- |
| N entering  at time-point | 159 | 149 | 130 | 114 | 99 | 89 | 72 | 53 |
| N of terminal events (MDS-UPDRS 3.15/16/17 contralateral subitems ≥2) | 0 | 16 | 12 | 12 | 4 | 5 | 4 | 8 |
| Proportion with terminal event* | 0% | 10.7% | 19.0% | 27.5% | 30.4% | 34.3% | 38.0% | 47.4% |
| N withdrawing (censored) | 10 | 3 | 4 | 3 | 6 | 12 | 15 | 45 |

* Kaplan-Meier (product limit) method

**Supplementary table 4.** Results of the linear mixed effects models to test the effect of time, sex, age at diagnosis, disease duration at enrollment on bradykinesia, rigidity and tremor scores on the dominant and non-dominant sides of tremor in 159 participants with tremor requiring treatment (TRT). The dominant side of tremor was the side initially more affected with tremor.

|  | Bradykinesia | | Rigidity | | Tremor | |
| --- | --- | --- | --- | --- | --- | --- |
|  | Dominant side | Non dominant side | Dominant side | Non dominant side | Dominant side | Non dominant side |
| Intercept, estimate (SE) | 7.281 (1.352) | -2.224 (1.274) | 3.275 (0.571) | 0.152 (0.546) | 5.108 (0.756) | -0.755 (0.649) |
| Time, estimate (SE) | 0.247 (0.058)*** | 0.513 (0.055)*** | 0.098 (0.026)*** | 0.183 (0.024)*** | -0.008 (0.036) | 0.205 (0.028)*** |
| Age, estimate (SE) | -0.014 (0.021) | 0.070 (0.020)*** | -0.020 (0.009)* | 0.009 (0.008) | -0.023 (0.012) | 0.018 (0.010) |
| Disease duration, estimate (SE) | 0.045 (0.024) | 0.011 (0.023) | 0.008 (0.010) | -0.005 (0.010) | 0.003 (0.013) | -0.003 (0.011) |
| Sex, estimate (SE) | -0.703 (0.394) | 0.316 (0.372) | 0.321 (0.166) | 0.409 (0.159)* | 0.248 (0.220) | 0.412 (0.189)* |

Note: The variable “Time” refers to follow-ups, once every year after baseline. The variable “Sex” was coded as 0 for females and 1 for males. Age and disease duration are considered at enrollment. Significance level: * p < 0.05; p < 0.01; p < 0.001

**Supplementary Figures**

**Supplementary Figure 1**. Diagram showing lower limb tremor distribution in the 397 idiopathic PD participants included in the study.

Abbreviations. UL: upper limbs. LL: lower limbs.

**Supplementary Figure 2.** Probability functions (estimated with the Kaplan-Meier method) for the development of TRT (i.e. a score equal or greater than 2 in any of the tremor subitems of MDS-UPDRS items 3.16, 3.17 or 3.18) in 119 participants with mild lateralized tremor at baseline, ipsilaterally (left panel) and contralaterally (right panel).


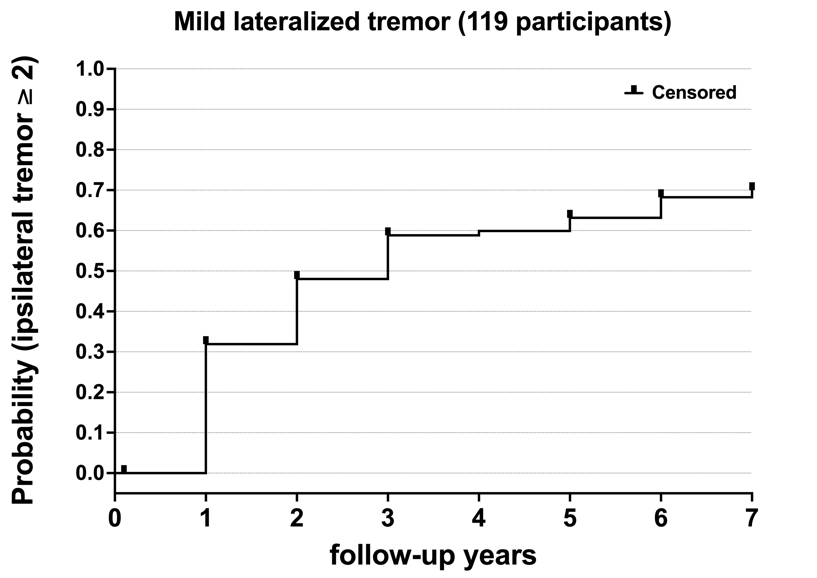

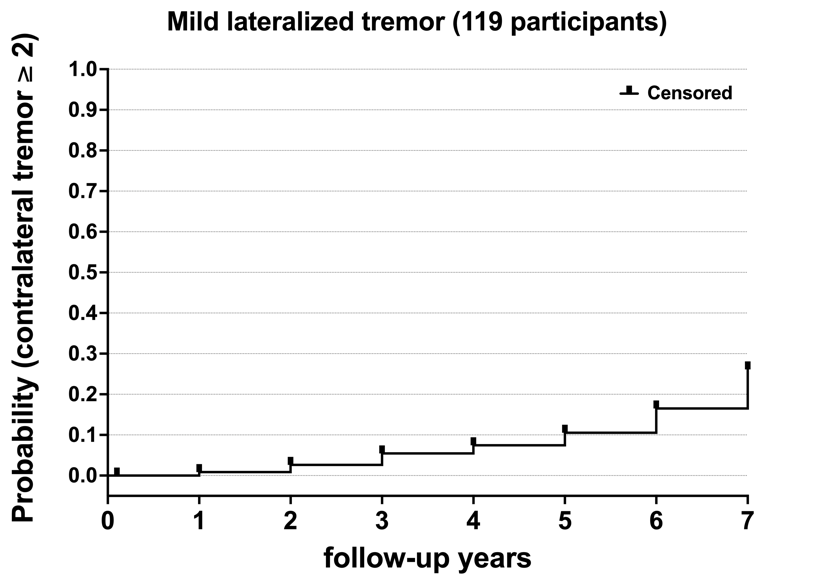

Supplement: Supplementary file 1 — TABLE S1. Number of participants with a lateralized tremor at baseline involved at each follow up visit with the off‐state assessment (A) and on‐state assessment (B). The number of total participants is also split by right‐dominant and left‐dominant. TABLE S2. Distribution of tremor types at baseline OFF‐condition in participants with a lateralized tremor. TABLE S3. Survival table related to the development of contralateral upper limbs tremor score ≥2 in 159 participants with lateralized tremor at baseline. TABLE S4. Results of the linear mixed effects models to test the effect of time, sex, age at diagnosis, disease duration at enrollment on bradykinesia, rigidity and tremor scores on the dominant and non‐dominant sides of tremor in 159 participants with tremor requiring treatment (TRT). The dominant side of tremor was the side initially more affected with tremor. Figure S1. Diagram showing lower limb tremor distribution in the 397 idiopathic PD participants included in the study. Figure S2. Probability functions (estimated with the Kaplan–Meier method) for the development of TRT (ie, a score equal or greater than two in any of the tremor subitems of MDS‐UPDRS items 3.16, 3.17 or 3.18) in 119 participants with mild lateralized tremor at baseline, ipsilaterally (left panel) and contralaterally (right panel). [file MDC3-13-702-s001.docx]
